# Supplementary material for: Markov modeling reveals novel intracellular modulation of the human TREK-2 selectivity filter
Source: Sci Rep. 2017 Apr 4;7:632. doi: 10.1038/s41598-017-00256-y (PMC5428000; doi:10.1038/s41598-017-00256-y)
Supplement: Supplementary file 2 — Supplementary Info [file 41598_2017_256_MOESM2_ESM.pdf]

Supporting Information for: Markov modeling reveals novel intracellular modulation of the human TREK-2 selectivity filter

Matthew P. Harrigan,[1,*] Keri A. McKiernan,[1,*] Veerabahu Shanmugasundaram,[2] Rajiah Aldrin Denny,[3,†] and Vijay S. Pande[1,4,5,6,‡]

[1]*Department of Chemistry, Stanford University, Stanford, CA 94305*

[2]*Medicinal Sciences, Pfizer Inc., Eastern Point Road, Groton, CT 06340*

[3]*Medicinal Sciences, Pfizer Inc., 610 Main Street, Cambridge, MA 02139*

[4]*Department of Computer Science, Stanford University, Stanford, CA 94305*

[5]*Department of Structural Biology, Stanford University, Stanford, CA 94305*

[6]*Program in Biophysics, Stanford University, Stanford, CA 94305*

[*] Denotes equal contribution

[†] Corresponding author: Aldrin.Denny@pfizer.com

[‡] Corresponding author: pande@stanford.edu

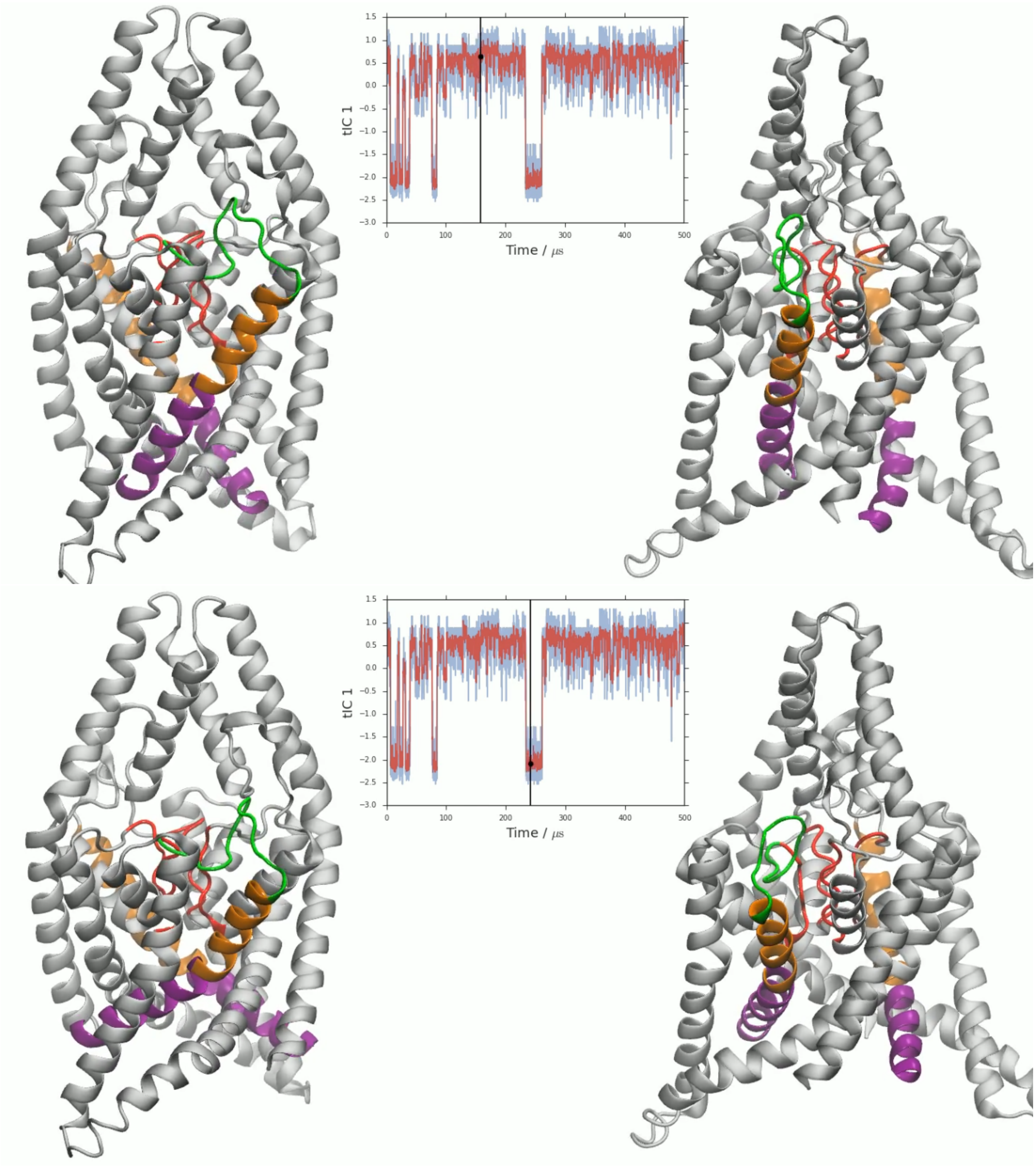

FIG. S1: We generated a 500 μs representative MSM trajectory by sampling among discrete states according to the modeled transition probabilities. The movie can be viewed in the supplementary file `trek.mp4`. Depicted here are two frames from the movie (top and bottom). Each frame consists of a view of the protein and its 90 degree rotation. We plot the projection of the conformation against its tIC 1 (Up-Down) coordinate. The black vertical line traces the current time in the trajectory.

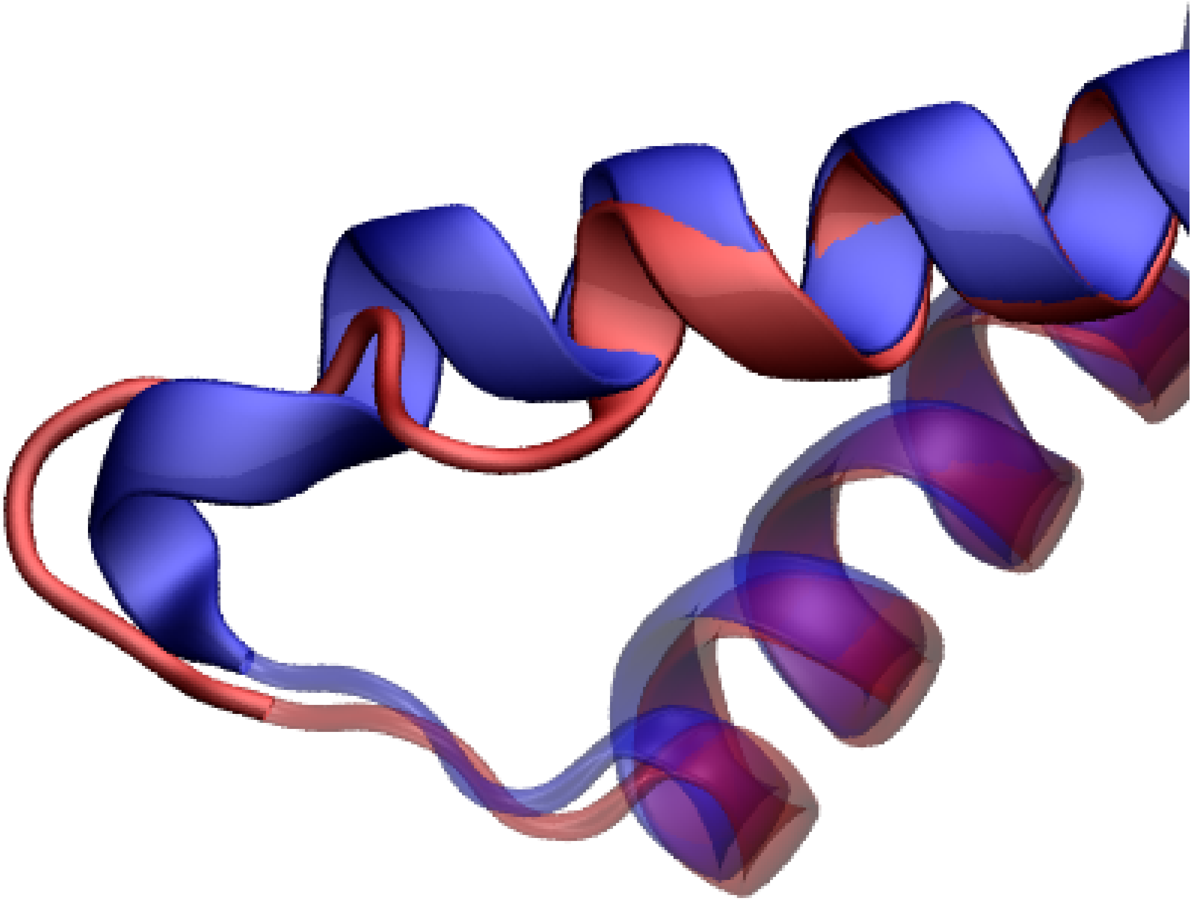

FIG. S2: Partial unfolding of the M2-M3 loop distinguishes I_2 from *Down*. Colors are as in Fig. 1B.

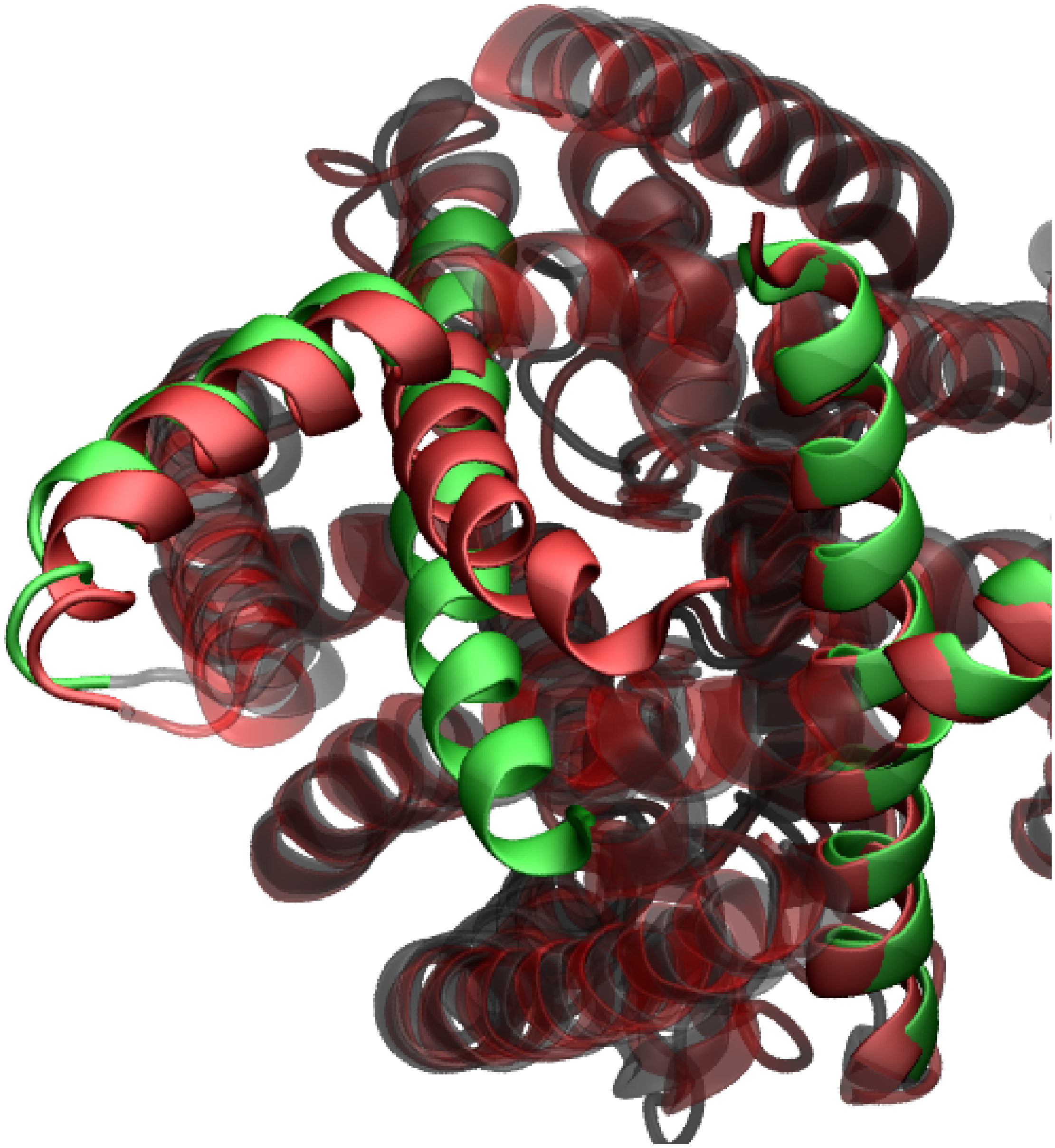

FIG. S3: I_1 and I_2 differ in positioning of the M4 helix. I_2 adopts the down-like configuration of this helix whereas I_1 adopts an an up-like configuration of this helix. Both intermediates adopt the down-like M2-M3 helix positions. From a purely structural standpoint, I_1 may be suggested to be "half down or half up". However, it is strongly kinetically related to the down conformations. Specifically, we observe a rapid relaxation from I_1 to I_2. Colors are as in Fig. 1B.

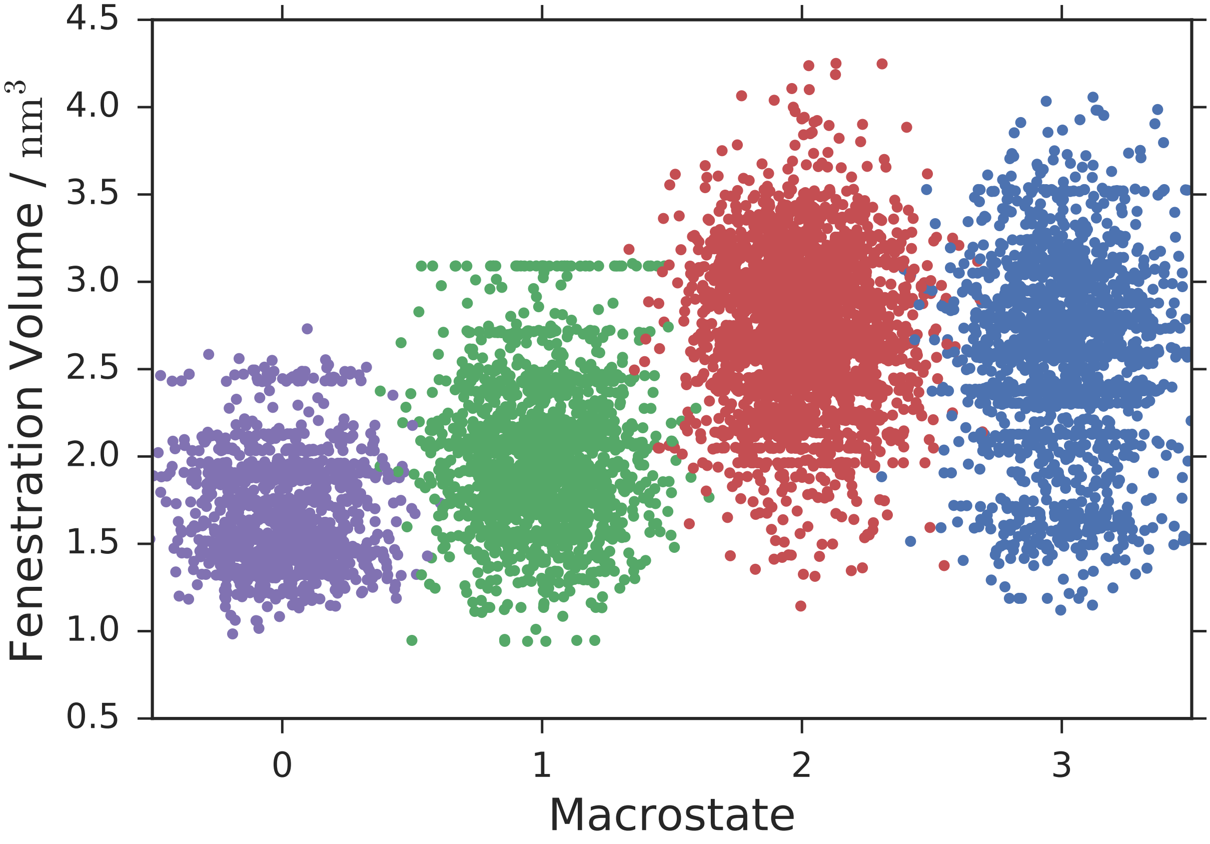

FIG. S4: Fenestration volume increases in macrostates I_2 and *Down*. Large fenestrations are created in the down conformations. Colors are as in Fig. 1B.

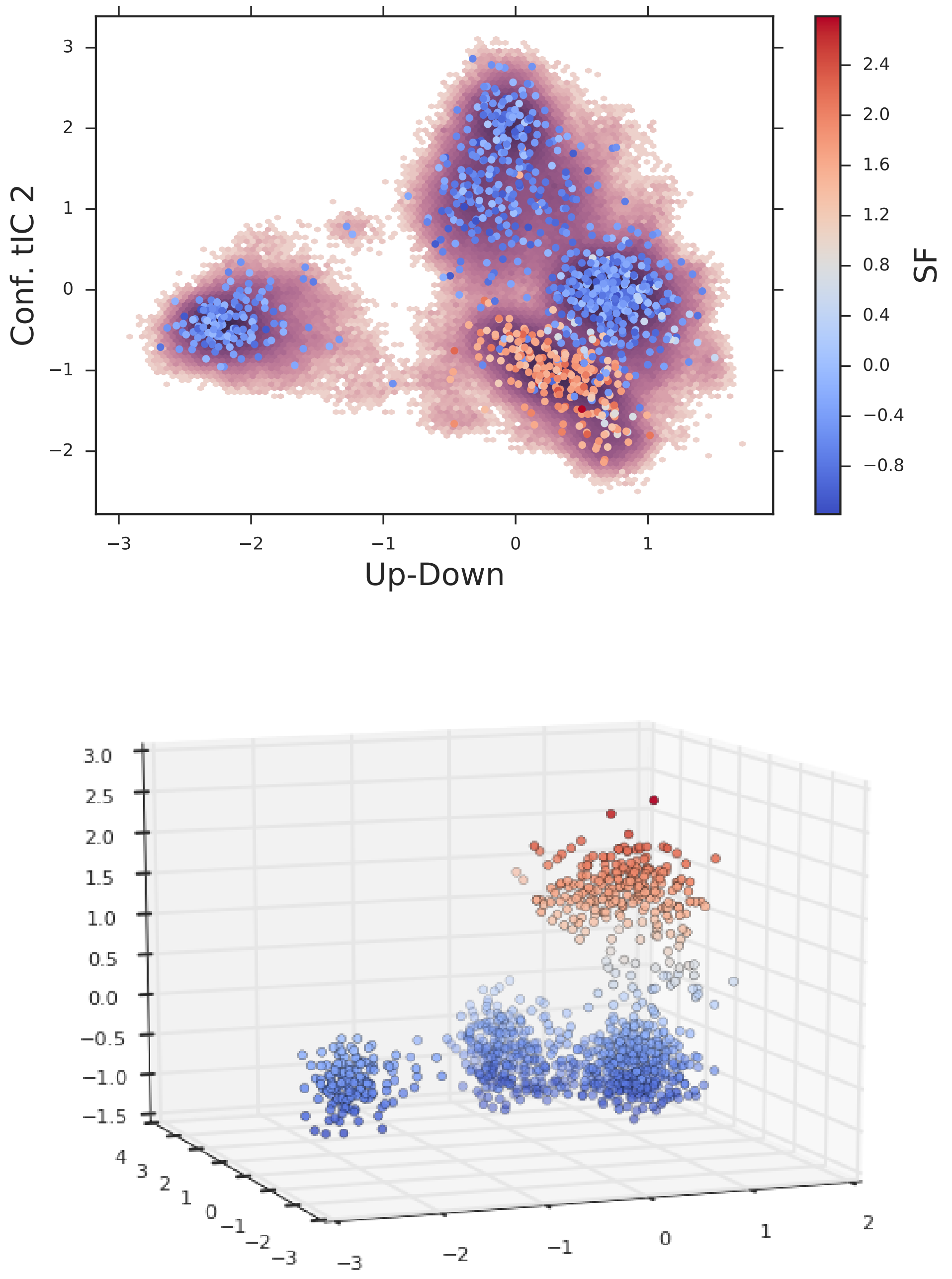

FIG. S5: We attempt to plot the three-dimensional histogram of observations versus the two conformational tICs and the SF tIC. (top) SF tIC value of cluster centers given by color. (bottom) 3D scatter plot of cluster centers, again colored by SF tIC value. These plots can be seen as a combination of Fig. 1B and C. The coupling is shown by the inset table in Fig. 1C.

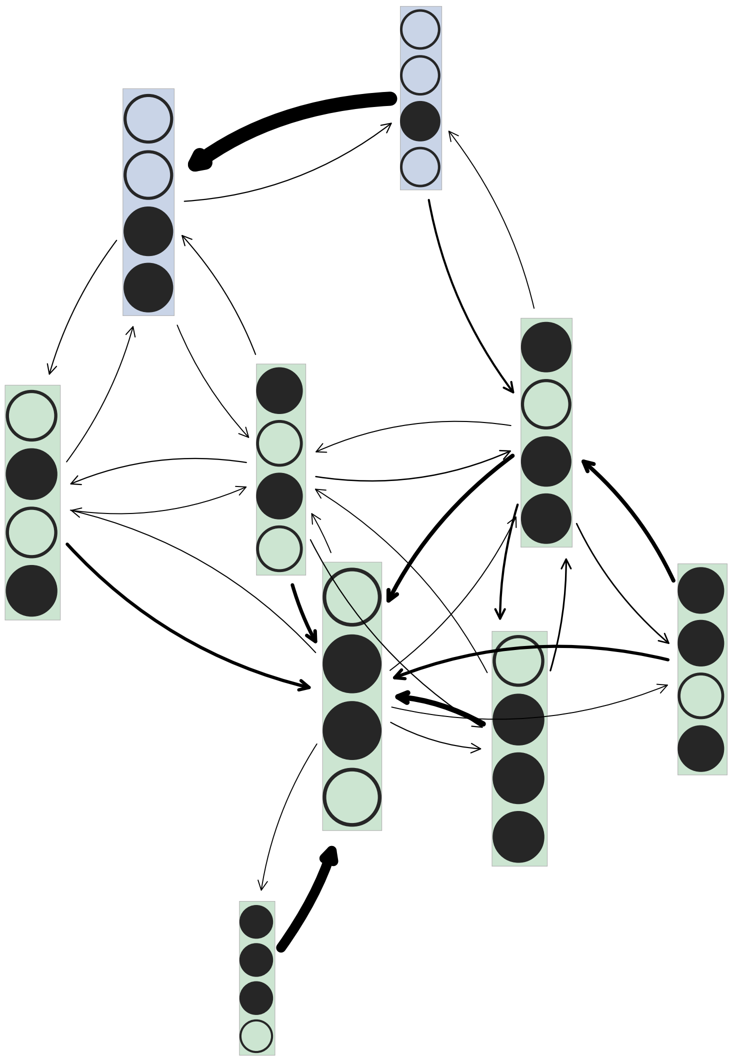

FIG. S6: Compare with Fig. 2 A and C. Whereas those figures show ion microstate transition graphs partitioned by *Up* and *Down* macrostates (resp), here we plot the microstate transition graph for the whole dataset without partitioning by macrostate.

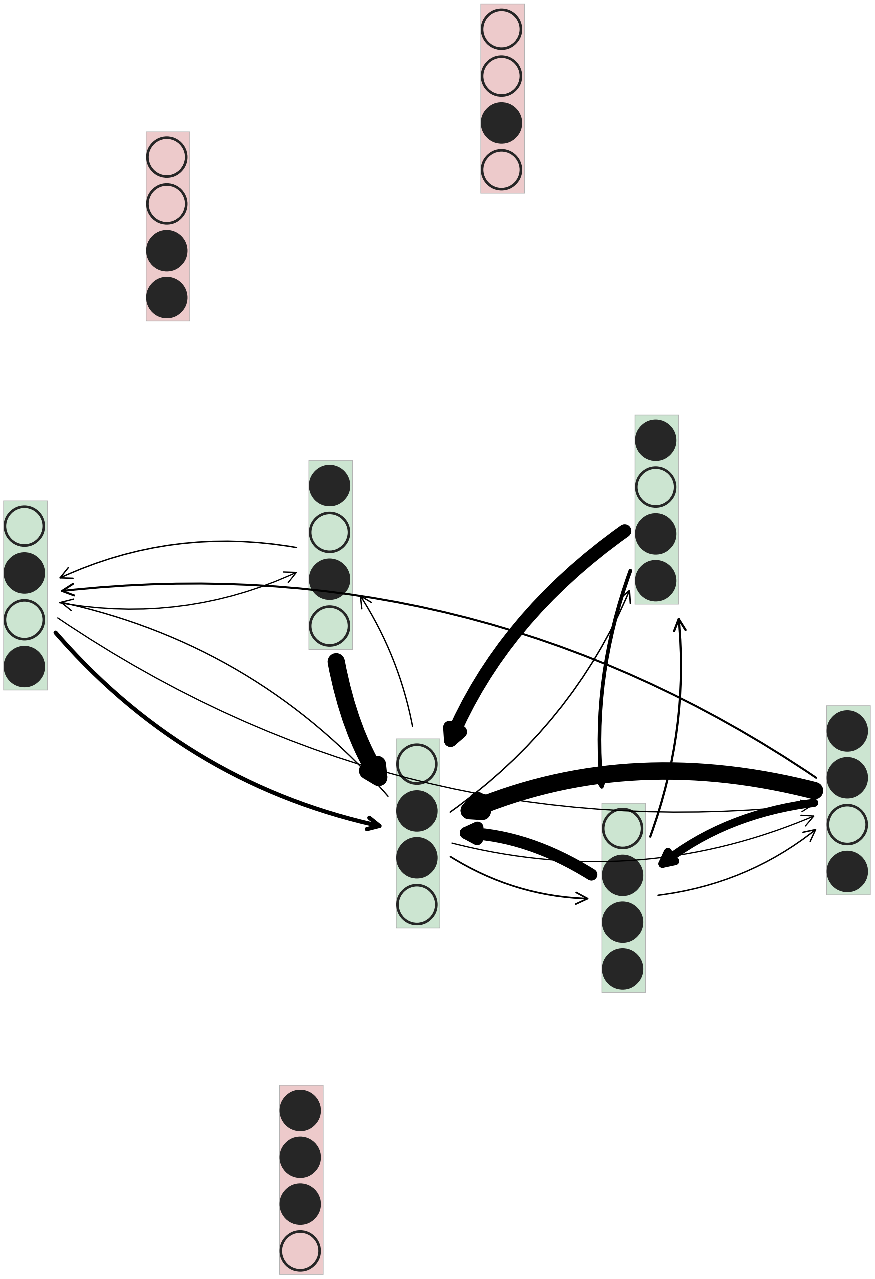

FIG. S7: Compare with Fig. 2 A and C. Whereas those figures show ion microstate transition graphs partitioned by *Up* and *Down* macrostates (resp), here we plot the microstate transition graph for the I_1 macrostate.

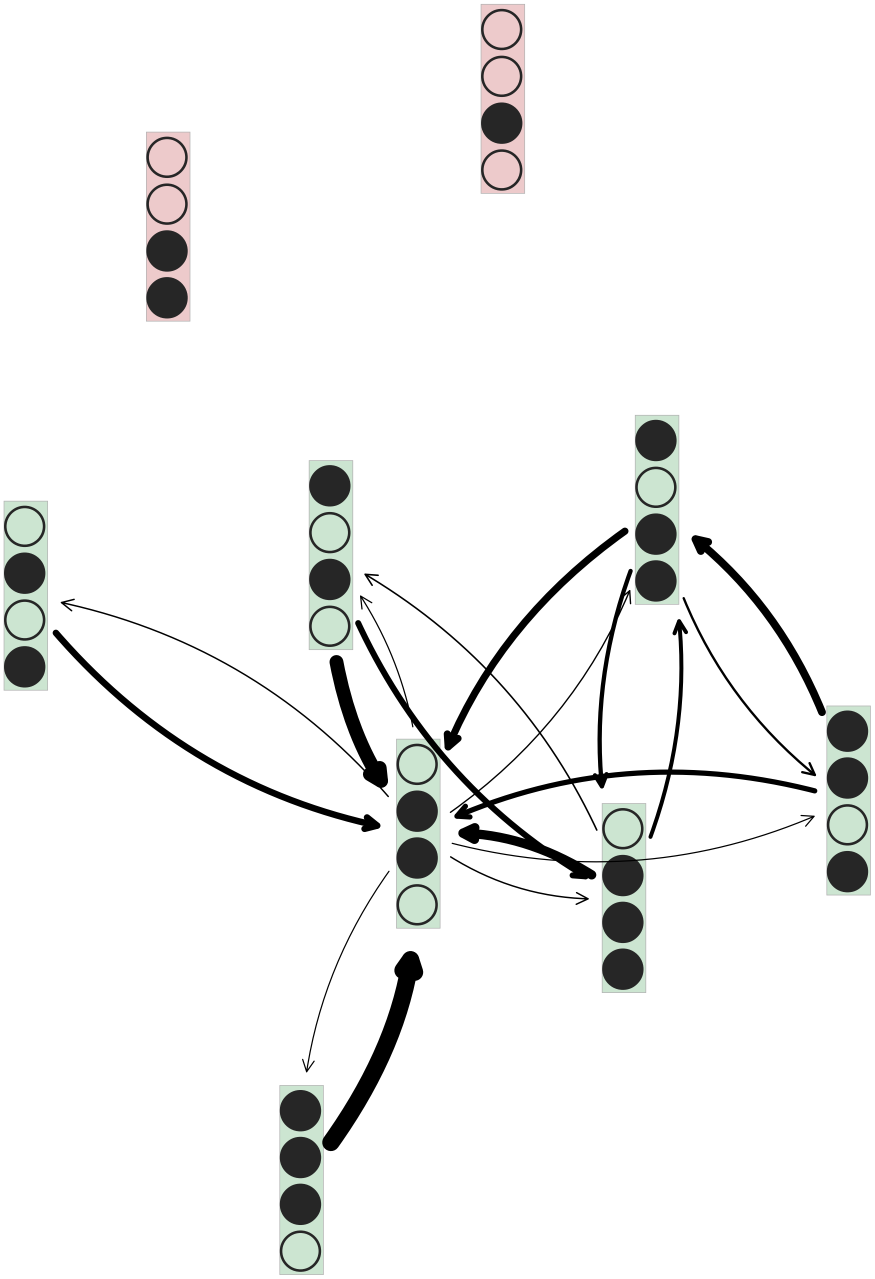

FIG. S8: Compare with Fig. 2 A and C. Whereas those figures show ion microstate transition graphs partitioned by *Up* and *Down* macrostates (resp), here we plot the microstate transition graph for the I_2 macrostate.
